# Genetic transmission networks of HIV-1 CRF07_BC strain among HIV-1 infections with virologic failure of ART in a minority area of China: a population-based study

**DOI:** 10.1186/s12879-020-05347-2

**Published:** 2020-08-26

**Authors:** Dan Yuan, Meijing Liu, Yiping Li, Peng Jia, Ling Su, Li Ye, Yan Zhang, Pan Ling, Chang Zhou, Shu Liang, Hong Yang, Honglu Liu, Shujuan Yang

**Affiliations:** 1 Center for AIDS/STD Control and Prevention, Sichuan Center for Disease Control and Prevention, Chengdu, China; 2 International Institute of Spatial Lifecourse Epidemiology (ISLE), Hong Kong, China; 3 West China School of Public Health and West China Fourth Hospital, Sichuan University, Number16, Section 3, South Renmin Road, Chengdu, 610041 Sichuan China; 4 Department of Land Surveying and Geo-Informatics, The Hong Kong Polytechnic University, Hong Kong, China

**Keywords:** HIV-1, Genetic transmission network, CRF07_BC, Virologic failure, Antiretroviral therapy

## Abstract

**Background:**

The drug resistance and the virologic failure of antiretroviral therapy (ART) are quite severe in Liangshan. A better understanding of the virologic failure of ART and the HIV-1 transmission network dynamics is essential for the surveillance and prevention of HIV. Here, we analyzed the HIV-1 CRF07_BC strain genetic transmission networks and their associated factors among people living with HIV-1 (PLWH) who had virologic failure of ART by using close genetic links.

**Methods:**

The drug-resistant mutations were determined using the Stanford University HIV Drug Resistance Database. HIV-1 *pol* genes sequences were used for phylogenetic and genotypic drug resistance analysis. The genetic transmission networks were performed by comparing sequences, constructing the phylogenetic tree, calculating the pairwise distance, and visualizing the network.

**Results:**

A total of 1050 PLWH with CRF07_BC *pol* sequences were finally identified and included in the genetic transmission network analysis from 2016 to 2017. Of the 1050 CRF07_BC *pol* sequences, 346 (32.95%) fell into clusters at a genetic distance of 0.006, resulting in 137 clusters ranging in size from 2 to 40 individuals. Subjects who were widowed or divorced were less likely to form a genetic transmission network (adjusted OR: 0.50), while subjects who had shared a needle ≥ five times were more likely to form a network (adjusted OR: 1.88).

**Conclusions:**

The genetic transmission networks revealed the complex transmission pattern, highlighting the urgent need for transmission monitoring of virologic failure of ART and selection of more effective therapeutic regimens to promote viral suppression.

## Background

The Human Immunodeficiency Virus /Acquired Immune Deficiency Syndrome (HIV/AIDS) epidemic has become one of the most critical issues that seriously affect public health. Since the start of the epidemic, around 75 million people have been infected with HIV, and in 2018, 37.9 million people were living with HIV (PLWH) worldwide [1]. By the end of October 2019, approximately 958 thousand people lived with HIV in China [2]. Liangshan Prefecture, as the autonomous prefecture with the largest population of Yi people in southwest China, contains a huge of a proportion of PLWH in Sichuan Province, where the prevalence of PLWH was over 1% in Butuo County, Zhaojue County, Meigu county, Yuexi County, and Jinyang County, ranking among the top 5 counties in the country. According to previous sampling survey reports, the effective rate of antiretroviral therapy (ART) among PLWHin Liangshan Prefecture was only 55.9%, and the drug resistance rate of newly reported untreated patients was as high as over 5% [3]. The high virologic failure rate and drug resistance rate of ART in Liangshan Prefecture were mainly due to the poor adherence to ART, the high mobility, and the high lost-to-follow-up rate of PLWH [4, 5]. Besides, Linda et al. [6, 7] found a more unsatisfactory virologic response in patients with transmitted drug resistance than those without. Therefore, we hypothesized that the main reason for high drug resistance rate in Liangshan Prefecture might be because of transmitted drug resistance before treatment. Thus, effective intervention should be formatted to reduce drug resistance in this area.

Three circulating recombinant forms (CRFs) of HIV-1 predominate in China, namely CRF01_AE, CRF07_BC, and CRF08_BC [8]. Subtype CRF07_BC was first discovered in the population with intravenous drug abuse in Yunnan Province and then rapidly spread to Xinjiang, Guangxi, and Sichuan Provinces [9]. According to a nationwide molecular epidemiological survey in 2006, HIV-1 CRF07_BC had become one of the most widespread subtypes circulating in China [10]. In Liangshan Prefecture, CRF07_BC was the main subtype of drug users in the past two decades and was slowly introduced to heterosexual people [11]. Now, CRF07_BC has become the most common form in this area [12, 13]. This study intends to construct an HIV-1 CRF07_BC strain transmission network of PLWH with virologic failure to analyze the clustering of drug-resistant strains and observe the possibility of drug-resistant transmission.

The HIV-1 molecular transmission network refers to a group of sequences that are not randomly gathered and have a certain epidemiological correlation. It constructs a transmission network through the genetic information of people infected with HIV through similar viruses with similarities and connections, and restores the macro social network of infected people as much as possible, which aims to focus on analyzing the characteristics of infected people, and preventing and controlling the active and critical groups in the network [14, 15]. HIV is transmitted through networks formed by closely connected individuals who engage in injecting or sexual behaviours [16, 17]. Understanding the structure and features of the transmission networks among HIV individuals with virologic failure can be useful for identifying potential transmission partners and recognizing the links between different populations [15], which is essential for developing HIV intervention strategies.

To our knowledge, few studies were performed to construct the HIV genetic transmission network with pol sequences identified from individuals with virologic failure of ART in China. Through the analysis of molecular transmission networks, we could obtain drug-resistant transmission clusters to provide policymaking recommendations for precious prevention for the virologic failure of ART. In this study, we investigated the HIV-1 CRF07_BC strain transmission patterns among PLWH who had virologic failure by using close genetic links and explored the factors associated with genetic transmission networks in Liangshan Prefecture.

## Methods

### Ethics statement

All PLWH voluntarily participated in our study and signed informed consent forms before enrollment. The study protocol was approved by the Ethics Committee of the Sichuan Center for Disease Control and Prevention, and the study was carried out following the Helsinki Declaration of 1964.

### Study participants

The inclusion criteria were: 1) being permanent residents or having stayed in the study sites for more than five years, 2) being confirmed with HIV-1 infection, 3) having received ART for more than six months, and 4) being confirmed to have a virologic failure of ART by the HIV RNA level.

Participants were recruited in Butuo county that had the most severe HIV epidemic of the Liangshan area. According to the basic information system for AIDS prevention and treatment, all PLWH of Butuo county in this system were included in our study.

**Fig. 1:**
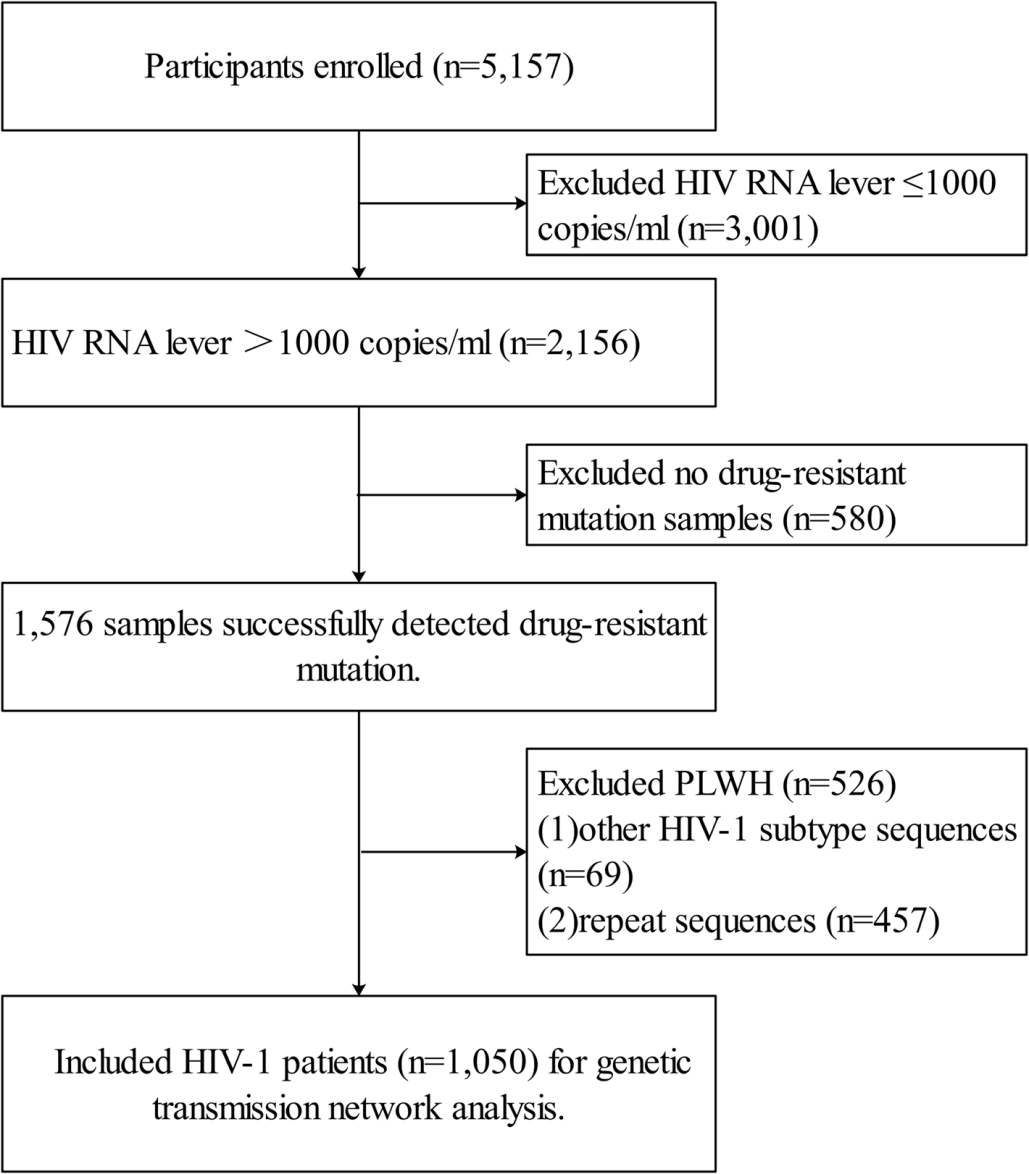
Sampling flowchart

A total of 5157 PLWH in Liangshan Prefecture were enrolled in this study. After excluding those with an HIV RNA level ≤ 1000 copies/ml, a total of 2156 PLWH showed virologic failure of ART. 1576 sequences were successfully obtained, and 1508 sequences of these were subtype CRF07_BC (95.69%). After excluding 69 other HIV-1 subtype sequences and 457 repeat sequences, a total of 1050 PLWH with CRF07_BC *pol* sequences were finally identified and included in the genetic transmission network analysis (Fig. 1).

### Data collection

The collected data included participants’ socio-demographic information, HIV related behaviours information, and ART related information. Socio-demographic information included age, occupation, marital status, attained level of education, treatment time, and self-reported route of HIV transmission (e.g., injection drug use, casual sexual behaviour, and risk behaviours related to mother-to-child transmission, etc.). Information on HIV related behaviours included needle sharing, the number of casual sexual partners, and the history of sexually transmitted diseases apart from HIV. ART related information, including the time of ART, the initial regimen of ART, change of ART regimen, and drug resistance, were extracted from their medical records.

### Laboratory tests

Each participant provided 5 mL of venous blood to detect the CD4^+^T cells and the viral load of HIV-1. The CD4^+^T cells were quantified within 12 h using flow cytometry at local county’s Center for Disease Control and Prevention (CDC). Plasma was isolated and sent on the cold chain to the Sichuan CDC to measure the HIV viral load. Virologic failure was defined as an HIV RNA level > 1000 copies/ml. For samples with an HIV RNA level > 1000 copies/ml, HIV drug resistance genotyping was conducted using an in-house PCR [18, 19].

### Nucleic acid extraction, amplification, and sequencing

RNA was extracted by an automatic extraction machine (MagNA Pure LC 2.0 system, Roche, Branchburg, NJ, USA) and the MagNA Pure LC Total Nucleic Acid Isolation Kit-High Performance kit (Roche, Branchburg, NJ, USA). The HIV-1 *Pol* region was amplified by two round nested polymerase chain reactions (Nested-PCR) using RNA extracted as a template. The amplification length was 1200 bp, including protease and reverse transcriptase regions.

The first-round PCR was performed by the AccessQuick RT-PCR System (A1702) kit (Promega Biotechnology Co., Ltd., Beijing, China), and the second-round PCR was performed by the Perfect shot TM Ex Taq Mix kit (Tiangen Biochemical Technology Co., Ltd., Beijing, China). The PCR products were treated with electrophoresis with 1% agarose gel. The amplified products were sent to the Beijing Genomics Research Center Ltd. for purification and gene sequencing. The detailed amplification and sequencing were described previously [19].

For samples with an HIV RNA level > 1000 copies/ml, the evaluation of drug resistance mutations was performed by using the algorithm of the Stanford HIV drug resistance database (http://hivdb.stanford.edu/). All experimental protocols followed the manufacturer’s instructions.

### HIV-1 genetic transmission network

The analysis of this step was described in our previous study [20]. By comparing sequences, constructing the phylogenetic tree, calculating the pairwise distance, and visualizing the network, the flow chart of the genetic transmission network was realized. To avoid the potential confounding effect of the convergent evolution of drug resistance, we constructed the genetic transmission network after excluding 32 codon positions in protease and reverse transcriptase associated with drug resistance.

After excluding repeat sequences, we performed the phylogenetic tree and molecular transmission networks based on pol sequences identified from individuals with virologic failure of ART. Calculations of the pairwise genetic distances of all sequences within the available clusters were performed by Run HyPhy 2.2.4 (http://www.hyphy.org). Within the genetic distances of 0.001–0.015, the gene distance with the largest number of clusters was selected as the linkage within a cluster. Finally, the network visualization analysis was based on the Cytoscape 3.5 network software (www.cytoscape.org) [20].

### Statistical analysis

Frequencies and percentages were used to represent the categorical variables, and the chi-square (χ^2^) test was used to compare the differences among groups. Taking the presence of individuals within the transmission networks as the dependent variable, unadjusted OR was estimated for the socio-demographic variables, HIV-related behaviours, ART-related information, the first CD4^+^ count after diagnosis, the stage of the diseases, and drug resistance. A binary regression model was fitted, and variables with significance in the univariate analysis were considered as candidate variables. SPSS version 21.0 for Windows (SPSS, Inc., Chicago, IL, USA) was used for data analysis, with *p* values< 0.05 taken as statistically significant.

## Results

### Distribution of HIV-1 genotypes among PLWH with drug resistance

Most of the subtypes of HIV-1 were CRF07_BC (1508, 95.62%), followed by the subtypes CRF08_BC (42, 2.66%), and C (27, 1.71%). Among PLWH with virologic failure of ART, the drug-resistant rate was as high as 32.10% (507/1576). We found that 477 (30.25%) subjects showed drug resistance to non-nucleoside reverse transcriptase inhibitors (NNRTIs), 193 (12.24%) to nucleoside reverse transcriptase inhibitors (NRTIs), and 29 (1.84%) to protease inhibitors (PIs) in ART.

### Characteristics of PLWH within the transmission network

A total of 1050 PLWH with CRF07_BC *pol* sequences were identified and included in the genetic transmission network analysis. A total of 346 PLWH were within the genetic transmission network with a genetic distance of 0.006. In the univariate analysis, using the presence of PLWH within the transmission networks as the dependent variable, PLWH with needle sharing behaviour were more likely to be included in the transmission networks than PLWH without drug use or sharing needles, and PLWH who were widowed or divorced were less likely to be included in the transmission networks than PLWH with marriage (Table 1).

**Table 1:** The distribution of PLWH with virologic failure in ART in the genetic transmission networks

	Genetic transmission network		*N* = 1050	Unadjusted OR (95% CI)
	*n* = 346	%		
**Socio-demographics**				
**Gender**				
Male	240	33.61	714	1.0 (Ref.)
Female	106	31.55	336	0.91(0.69–1.20)
**Age, years**				
≤ 15	34	33.66	101	1.0 (Ref.)
15 ~ 40	240	32.92	720	0.98(0.63–1.53)
>40	72	31.44	229	0.90(0.55–1.49)
**Occupation**				
Employed	12	28.57	42	1.0 (Ref.)
Peasant	297	33.07	898	1.24(0.62–2.45)
Students and children	28	35.90	78	1.40(0.62–3.16)
Unknown	9	28.13	32	–
**Current marital status**				
Married	263	35.44	742	1.0 (Ref.)
Single	63	28.38	222	0.72(0.52–1.02)
Widowed or Divorced	11	21.57	51	0.50(0.25–0.99) **
Unknown	9	25.71	35	–
**Ethnicity**				
Han and others	2	25.00	8	1.0 (Ref.)
Yi	344	33.01	1042	1.48(0.30–7.36)
**Education level**				
Illiteracy	221	31.80	695	1.0 (Ref.)
Primary school	116	36.59	317	1.24(0.94–1.64)
Secondary or above	9	23.68	38	0.67(0.31–1.43)
**HIV transmission route**				
Heterosexual behaviors	123	32.11	383	1.0 (Ref.)
Drug injection	139	34.58	402	1.12(0.83–1.50)
Heterosexual behaviors and drug injection	49	30.63	160	0.93(0.63–1.39)
Mother to child	35	33.33	105	1.06(0.67–1.67)
**CD4**^**+**^ **cell count, cell/μl**				
≤ 200	63	38.65	163	1.0 (Ref.)
201 ~ 500	174	31.02	561	0.71(0.50–1.03)
>500	101	33.89	298	0.81(0.55–1.21)
Unknown	8	28.57	28	–
**Viral load, copies/ml**				
1000 ~ 10,000	175	33.72	519	1.0 (Ref.)
10,000 ~ 50,000	101	31.96	316	1.05(0.75–1.48)
>50,000	70	32.56	215	0.97(0.67–1.41)
**Stage of disease**				
HIV	143	32.43	441	1.0 (Ref.)
AIDS	172	33.79	509	1.06(0.81–1.40)
Unknown	31	31.00	100	–
**Time of diagnosis of HIV, years**				
< 3	69	36.51	189	1.0 (Ref.)
3–5	71	36.22	196	0.99(0.65–1.50)
> 5	206	30.98	665	0.78(0.56–1.10)
**HIV related behaviors**				
**Needle sharing**				
Had never used illicit drugs or shared a needle	177	31.72	558	1.0 (Ref.)
Had shared a needle 1 ~ 4 times	91	30.95	294	0.97(0.71–1.31)
Had shared a needle ≥5 times	78	39.39	198	1.40(1.01–1.96) **
**Number of casual sexual partners**				
0	199	32.52	612	1.0 (Ref.)
1 ~ 4	92	34.72	265	1.10(0.81–1.50)
≥ 5	55	31.79	173	0.97(0.67–1.39)
**History of sexually transmitted diseases except for HIV**				
Never	258	31.81	811	1.0 (Ref.)
Ever	88	36.82	239	1.25(0.92–1.69)
**ART related information**				
**Time of ART, years**				
≤ 12	67	40.85	164	1.0 (Ref.)
12 ~ 36	127	32.90	386	0.71(0.49–1.04)
>36	100	32.47	308	0.70(0.47–1.04)
Unknown	52	27.08	192	–
**Initial regimen of ART**				
TDF + 3TC + EFV/NVP	217	35.11	618	1.0 (Ref.)
AZT + 3TC + EFV/NVP	63	31.66	199	1.17(0.83–1.64)
LPV + 3TC + AZT/TDF	13	33.33	39	1.08(0.52–2.24)
Unknown	53	27.32	194	–
**Change of ART regimen**				
Never	263	34.07	772	1.0 (Ref.)
Ever	31	36.05	86	1.09(0.69–1.74)
Unknown	52	27.08	192	–
**Drug resistance (DR)**				
No	228	31.58	722	1.0 (Ref.)
Yes	118	35.98	328	1.22(0.89–1.60)
**DR to NNRTI**				
No	236	31.85	741	1.0 (Ref.)
Yes	110	35.60	309	1.18(0.89–1.56)
**DR to NRTI**				
No	302	32.83	920	1.0 (Ref.)
Yes	44	33.85	130	1.05(0.71–1.54)
**DR to PI**				
No	343	33.20	1033	1.0 (Ref.)
Yes	3	17.65	17	2.32(0.66–8.13)

Unadjusted OR: Unadjusted odds ratios

**P value< 0.01

In the summary of the binary logistic regression model, we found that PLWH who were widowed or divorced were less likely to form a genetic transmission network (adjusted OR: 0.50, 95%CI: 0.25–0.99; reference group: married), while PLWH who had shared a needle ≥5 times were more likely to be in a transmission network (adjusted OR: 1.88, 95%CI: 1.02–3.46; reference group: had never used illicit drugs or shared a needle) (Table 2). The ratio of men to women being widowed or divorced was 4:7, while the ratio was 6:1 among singles (Data not shown).

**Table 2:** Factors associated with the presence of PLWH within the genetic transmission networks

	Adjusted OR (95% CI)
**Current marital status**	
Married	1.0 (Ref.)
Single	0.72(0.52–1.01)
Widowed or Divorced	0.50(0.25–0.99) **
Unknown	–
**Needle sharing**	
Had never used illicit drugs or shared a needle	1.0 (Ref.)
Had shared a needle 1 ~ 4 times	1.28(0.72–2.27)
Had shared a needle ≥5 times	1.88(1.02–3.46)**

Adjusted OR: Adjusted odds ratios, variables listed in Table 1 with *p* < 0.05 in the univariate analysis as candidates were selected by a summary multiple logistic regression model

**P value< 0.01

### HIV-1 genetic transmission network

**Fig. 2:**
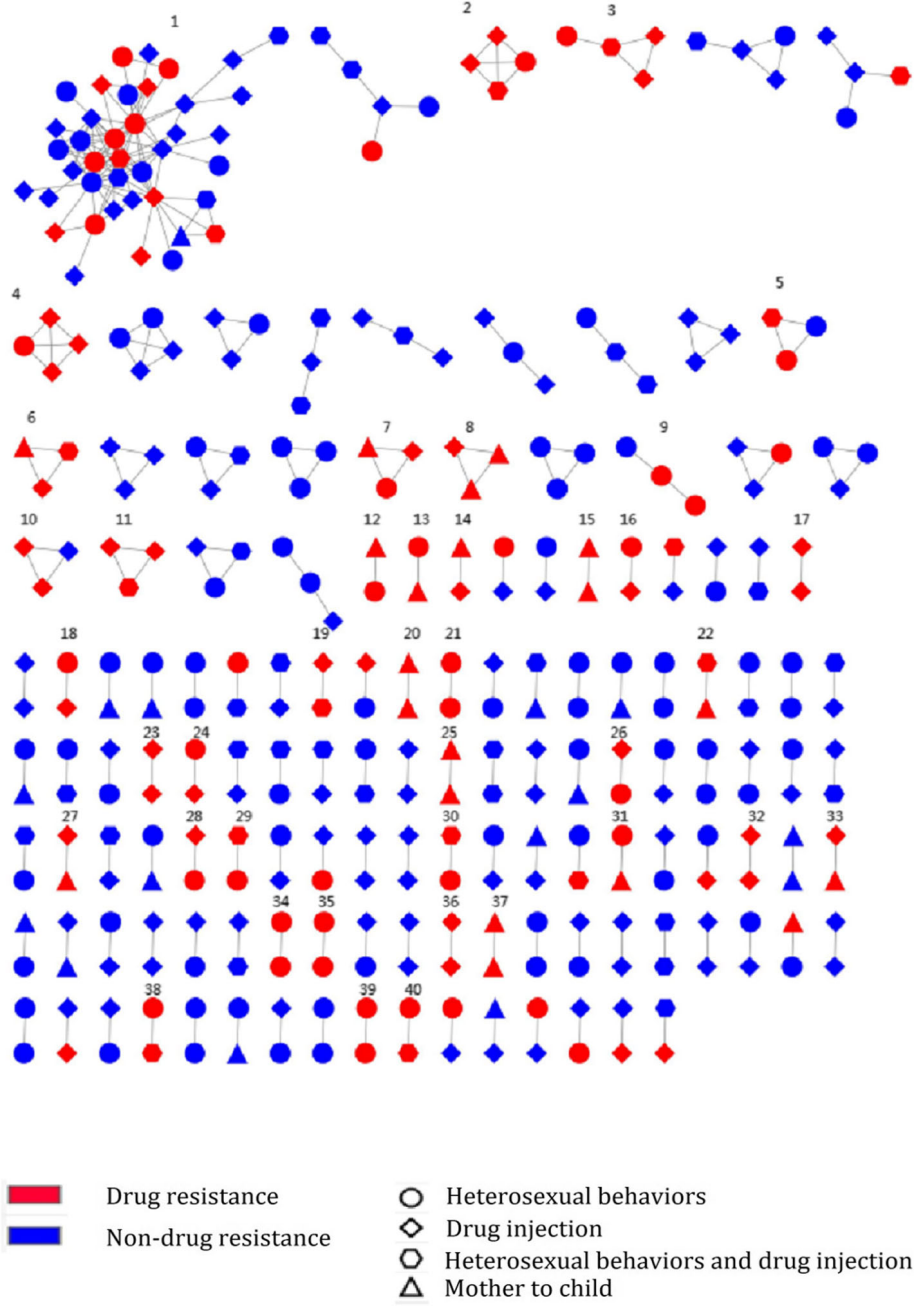
Genetic transmission networks by drug resistance. 1–40 are transmission clusters containing drug-resistant sequences

A total of 137 clusters were observed with the number of sequences per cluster ranging from 2 to 40. Of these clusters, 107 clusters had only two linked PLWH (one link), and 30 clusters had ≥2 potential transmission partners (Fig. 2).

**Fig. 3:**
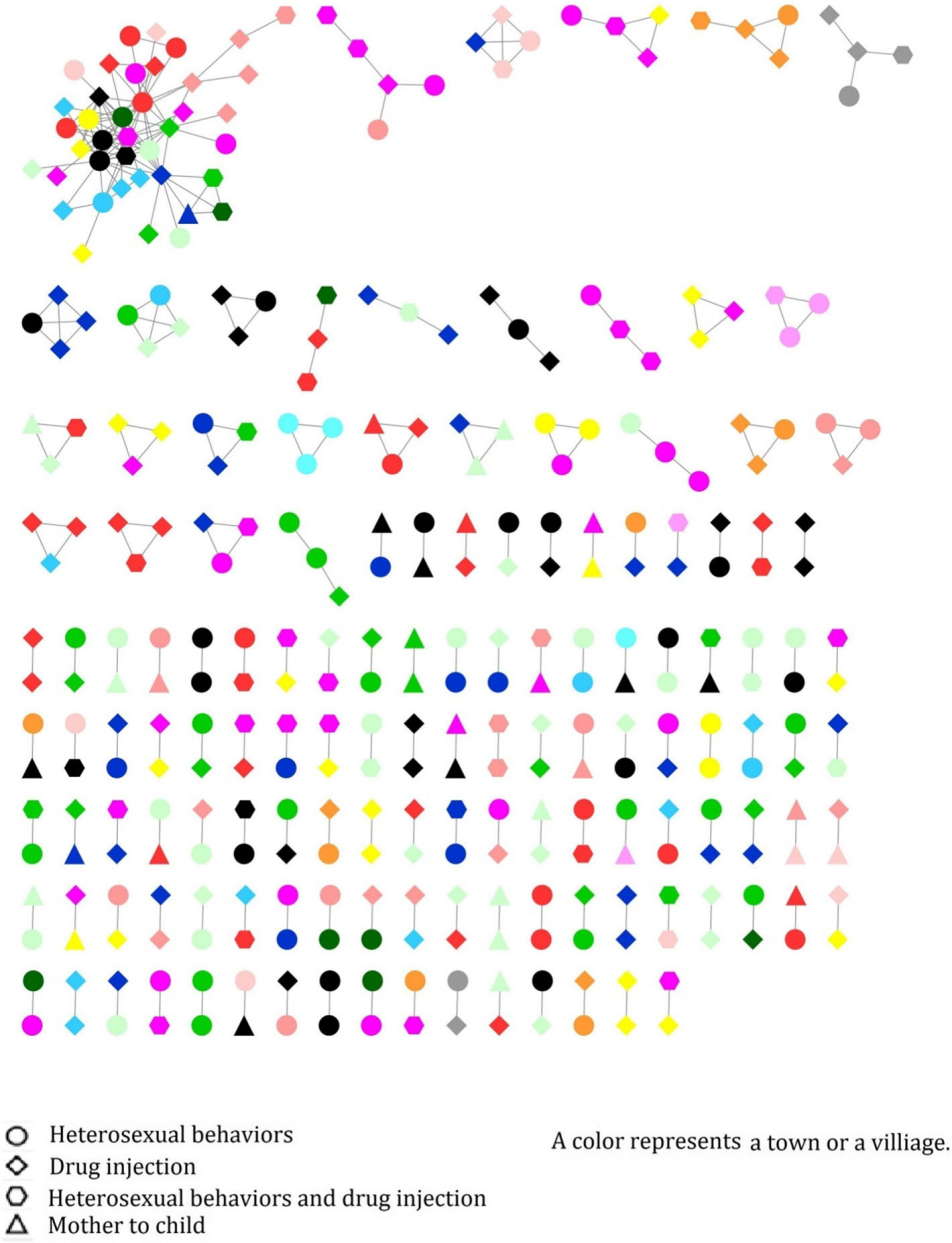
Genetic transmission networks by places of residence

A total of 118 drug resistance sequences were observed in the transmission network. Among these sequences, 101 sequences (85.59%) were linked to at least one other drug resistance sequence, and 40 clusters had > 2 drug resistance sequences, indicating that PLWH with drug resistance were more likely to link with those with drug resistance (Fig. 2). Among these 40 clusters, 39 clusters (97.5%) had common drug resistance sites, 22 clusters (55%) shared the common site of K103N, 16 clusters (40%) shared the common site of M184V/I, and 9 clusters (22.5%) shared the common site of Y181C, which suggested that the linked sequences with drug resistance tended to have common drug resistance sites (Fig. 2, Additional file 1). On the HIV transmission routes, we found that drug injection and heterosexual contact were often cross-linked (Fig. 3). Moreover, 51.54% (50/97) of the clusters with two PLWH, 57.89% with 3–4 PLWH, and all clusters with more than 5 PLWH were from different villages or towns (Fig. 3).

## Discussion

Liangshan Prefecture is the largest traditional settlement of the Yi minority people in China and is one of the areas in China severely affected by HIV [21]. In Liangshan, the rapid scale-up of ART led to a significant decline in morbidity and mortality among PLWH [22]. However, the virologic failure of ART and drug resistance are still serious in this area. Therefore, monitoring the virologic failure of ART and understanding the HIV-1 transmission network dynamics among patients with virologic failure of ART are urgently needed.

We found that a large number of sequences of drug resistance to ART were identified in the genetic transmission networks through the genetic transmission network analysis. In terms of drug resistance-associated mutations in transmission clusters, 35.98% of the individuals carrying drug resistance were involved in the network. 101 PLWH (85.59%) with drug resistance were linked with each other, which suggested the possibility of drug resistance transmission. Moreover, most of the linked sequences had common drug resistance sites. Among 40 drug resistance clusters, K103N was observed in 22 clusters as the common drug resistance site and was the most frequent drug resistance-associated mutation. The possible reason might be that K103N mutation can persist without drug pressure and promote infection to another host [23, 24], which has been attributed to the widespread use of NNRTI in first-line ART [25]. Similar to K103N, M184V/I and Y181C can also be stably passaged without drug pressure [26] which may be the reason for the higher frequency of these drug resistance mutations in the transmission network. This finding was also observed in other undeveloped [25] and developed settings [27, 28]. The widespread use of ART may have led to a considerable increase in HIV drug resistance and drug resistance transmission in newly infected individuals. Drug resistance transmission may have potentially compromised the efficacy of the combination of ART regimens and may lead to failure of ART [29, 30]. Thus, there is an urgent need for routine resistance testing before starting treatment since the transmission of drug resistance was associated with virologic failure in patients [6, 7]. Also, close linkages between drug-resistance strains may be due to the high mutation of similar strains in the cluster, indicating that the ART regimen of PLWH within the cluster should be replaced if the drug-resistant mutations occurred in the same cluster.

Furthermore, our study indicated that most PLWH in the clusters were from different villages or towns. Unlike other minority groups in China, the Yi minority people in Liangshan Prefecture are more likely to have casual sexual behaviours [21, 31, 32]. Casual sex often involves condomless sexual behaviours and multiple sex partners, facilitating the transmission of HIV [31, 32]. With the progress of society, the network has become more developed, and the population mobility has increased, which may increase the risk of the spread of HIV-1 and drug resistance transmission. Therefore, we should enhance health education on HIV-related behaviours for patients, especially the floating population, to reduce HIV-1 transmission.

We also found that widowed or divorced PLWH were less likely to link with other partners with genetically linked viruses. Most of the widowed or divorced PLWH were women (the men to women ratio were 4:7). These women may have been infected with HIV by their husbands through heterosexual transmission [33]. Because of the local social norms, widowed or divorced Yi women rarely have casual sex and extramarital sexual behaviours, making it challenging to spread HIV to others [21]. Therefore, effective and tailor-made programs are needed to reduce sexual transmission among unmarried youth and HIV-1 discordant couples.

### Limitations

Four limitations should be highlighted. First, this study was a cross-sectional survey, time-based sequences and cause-effect relationships among these variables cannot be established. Second, many factors, such as the complication of ART, the number of sexual partners, insurance status, and socioeconomic status should be considered as potential factors for influencing drug resistance and transmission networks. However, we could not obtain detailed epidemiological information about the sequences that were in the clusters. Further, follow-up medical records are warranted to include more epidemiological information. Third, the phylogenetic analysis is limited by the impossibility of inferring the direction of transmission, and genetic linkage could not reflect direct transmission due to lack of behavioural information of participants [34]. Fourth, since we did not include the sequences of all PLWH for analysis, we could not draw a whole transmission network, which limited the representation of the PLWH in this area.

## Conclusions

To our knowledge, we were the first to investigate the risk factors of transmission networks among HIV patients with virologic failure of ART in Liangshan. Our findings have enhanced the understanding of the HIV-1 genetic characteristics and transmission networks. Understanding the determinants of the genetic transmission network may contribute to designing preventive interventions for preventing HIV-1 transmission. However, it also highlighted the urgent need to monitor the transmission of virologic failure of ART and conduct effective therapeutic regimens to promote viral suppression.

## Supplementary information

**Additional file 1.** Analysis of drug resistance sites in clusters containing two or more drug resistance sequences

## Data Availability

The quantitative data used to support the findings of this study were supplied by the Center for AIDS/STD Control and Prevention, Sichuan Center for Disease Control and Prevention under license, and so cannot be made freely available. Requests for access to these data should be made to Shujuan Yang, rekiny@126.com.

## Abbreviations

HIV: Human Immunodeficiency Virus
AIDS: Acquired Immune Deficiency Syndrome
PLWH: People were living with HIV
ART: Antiretroviral therapy
CRFs: Circulating recombinant forms
DR: Drug resistance
