# Supplementary material for: Genetic transmission networks of HIV-1 CRF07_BC strain among HIV-1 infections with virologic failure of ART in a minority area of China: a population-based study
Source: BMC Infect Dis. 2020 Aug 26;20:631. doi: 10.1186/s12879-020-05347-2 (PMC7448482; doi:10.1186/s12879-020-05347-2)
Supplement: Supplementary file 1 — Additional file 1. Analysis of drug resistance sites in clusters containing two or more drug resistance sequences [file 12879_2020_5347_MOESM1_ESM.pdf]

Table S1 Analysis of drug resistance sites in clusters containing two or more drug resistance sequences

Cluster	Sequences	Common drug resistance sites	Non-common drug resistance sites
1	13	-	D67DN, M184M/V, K103N, E138EA, Y181YC, Y188YFHL/YH, V179D
2	4	T215S	-
3	4	K103N	-
4	4	K103N	-
5	2	K103N	-
6	3	M41L, V75I, M184V, T215F, V106M, F227L	-
7	3	M184V, Y181C	T215TNSY, H221HY
8	3	M184V, K103N	M230ML, K101KE, G190GA
9	2	K103N, V179E	-
10	2	K103N	-
11	3	K101E, Y181C	K103KN
12	2	M184V, K103N	T215FS
13	2	K103N	-
14	2	M184V, Y188L	-
15	2	G190A	-
16	2	K103N	K101KE
17	2	K103N	-
18	2	Y181C	-
19	2	K65R, L74I, M184V, L100I, K103N	M41ML, D67DN, V108I
20	2	M184V, V179E, Y181C	-
21	2	K103N	-
22	2	M184V, K103N, E138Q	-
23	2	M184I, K103N, P225H	-
24	2	K103N	-
25	2	M184V, K101E, G190A	-
26	2	M184V, V106M, V179D, G190A	K65KR, D67DN, K101E
27	2	E138G	-
28	2	Y181C	-
29	2	K103N	-
30	2	L74I, M184V, A98G, K103N, P225H	-
31	2	K103N	-

32	2	M184V, A98G, K101E, V108I, Y181C, G190A	-
33	2	K103N	-
34	2	D67N, K70R, M184V, T215F, K219E, Y181C	-
35	2	K103N	-
36	2	Y181C	-
37	2	M184V, L210LW, T215Y, Y181C	M41L, E44ED
38	2	K103N	-
39	2	M184V	K101E
40	2	K103N	-
